# Efficacy of inguinal buffered lidocaine and intranasal flunixin meglumine on mitigating physiological and behavioral responses to pain in castrated piglets

**DOI:** 10.3389/fpain.2023.1156873

**Published:** 2023-06-06

**Authors:** Magdiel Lopez-Soriano, Victoria Rocha Merenda, Stephanie Anderson, Pedro Henrique Esteves Trindade, Martin S. Leidig, Kristen Messenger, Juliana Bonin Ferreira, Monique Danielle Pairis-Garcia

**Affiliations:** ^1^ Department of Population Health and Pathobiology, College of Veterinary Medicine, North Carolina State University, Raleigh, NC, United States; ^2^ Department of Molecular Biomedical Sciences, College of Veterinary Medicine, North Carolina State University, Raleigh, NC, United States; ^3^ Graduate Program in Anesthesiology, Medical School, São Paulo State University (Unesp), Botucatu, Brazil; ^4^ Veterinary Practitioner, Mulfingen, Germany

**Keywords:** swine, local anesthetic, non-steroidal anti-inflammatory drug (NSAID), cortisol, castration, pain scale, animal welfare, Unesp-Botucatu pig composite acute pain scale (UPAPS)

## Abstract

Managing castration pain on US sow farms is hindered by the lack of Food and Drug Administration (FDA) approved products for mitigating pain. Previous work assessing flunixin meglumine (FM) efficacy in mitigating castration pain has shown the drug to be effective in pigs, meanwhile, results from previous work evaluating lidocaine efficacy are contradictory. Therefore, the objectives of this study were to determine the efficacy of inguinal buffered lidocaine (BL) and FM in mitigating castration pain in piglets. This study was divided into Part I (physiological response) and Part II (behavioral response). For part I piglets were randomly assigned to the following treatments: T1: (C) Castration plus physiological saline; T2: (S) Sham plus physiological saline; T3: (CL) Castration plus BL; T4: (SL) Sham plus BL; T5: (CF) Castration plus FM; T6: (SF) Sham plus FM; T7: (CLF) Castration plus BL and FM; T8: (SLF) Sham plus BL and FM. Blood was collected 24 h prior to castration, 1 h, and 24 h post castration for cortisol quantification. For Part II another cohort of piglets was enrolled and randomly assign to the following treatments: T1: (C) Castration plus physiological saline and T7: (CLF) Castration plus BL and FM. Behavior scoring was obtained in real-time by observing each piglet for 4-min continuously using Unesp-Botucatu pig acute pain scale (UPAPS) at the following timepoints: 1 h before castration (−1 h), immediately post-castration (0 h), and 3 h post-castration (+3 h). Average cortisol concentrations did not differ at −24 h (*P *> 0.05) or at 24 h post-castration (*P *> 0.05) between treatments. At 1 h post-castration, castrated piglets (C and CL) demonstrated greater cortisol concentrations (*P* < 0.05). Castrated piglets in the CF and CLF group had lower cortisol concentrations compared to C and CL-treated pigs (*P *< 0.05). For behavioral response, there were no differences between treatments on total UPAPS scores (C and CLF, *P* > 0.05). Intranasal FM was able to effectively reduce the physiological piglet's response immediately post-castration. Inguinal buffered lidocaine had no effect on the either physiological or behavioral response to pain. Long-term research should focus on refining injection techniques for inguinal BL and consider administration frequency and dosing of intranasal FM to control pain for a longer period post-castration.

## 1. Introduction

Castration is a painful procedure performed on awake piglets around the world without sedation or anesthesia intervention. In the US alone, more than 60 million of pigs are surgically castrated annually (1). Castration results in the piglet experiencing acute pain and stress (2, 3) and this procedure negatively impacts farm performance as demonstrated by increases in morbidity and mortality during the pre-wean production period (4, 5). Managing castration pain on US sow farms is hindered by two main drivers: (1) lack of Food and Drug Administration (FDA) approved products validated for efficacy in mitigating pain and (2) logistical limitations to implementing pain management protocols on a large scale (6).

In the US, relieving pain in pigs can be prescribed by veterinarians under the Animal Medicinal Drug Use Clarification Act (AMDUCA). This act permits veterinarians to utilize FDA-approved products in an extra-label manner (i.e., species and conditions not on the label), thus providing some options for pain relief while the US swine industry awaits approval of pain-specific products for pigs (7). As opportunities arise to approve products for pain relief, pharmaceutical companies should prioritize products that are effective, easy to administer, require minimal training and are as least invasive as possible, to overcome the logistical limitations found on large commercial farms.

Historically, lidocaine has been used on food animal species to inhibit pain transmission via local anesthesia (8). Lidocaine works primarily by blocking voltage-gated sodium channels thus inhibiting action potential propagation (9). Local anesthetics administration prior to castration is required in many European countries including Denmark, where veterinarians train caretakers to administer procaine, making the process more practical (10). However, results from previous work evaluating lidocaine efficacy for pain mitigation are contradictory. Some work suggests that intra-testicular administration of lidocaine mitigates pain (11–13), while other studies indicate that lidocaine does not effectively control post-operative castration pain (14, 15). In addition, lidocaine can reach peak concentrations around 3 min after administration (16) producing pain relief during the surgical procedure, however, it cannot control pain caused by inflammation from tissue damage after the castration process.

Currently, in the US, Flunixin meglumine (FM) is the most common pain relief used on swine farms (17). Flunixin meglumine is a non-steroidal anti-inflammatory drug (NSAID) that inhibits cyclooxygenase production and suppresses prostaglandin synthesis (18). This product can be administered via multiple routes including intramuscular, intravenous, topical, and oral (19). Previous work assessing FM efficacy in mitigating castration pain has shown the drug to be effective in pigs and other farm animal species undergoing castration (20–24).

Transdermal Flunixin meglumine was effective in mitigating pain in castrated pigs (24), suggesting its use as a pharmaceutical option to control pain in large commercial farms given its advantage as a non-invasive, extra-label administration route. To the author's knowledge, no studies to date have evaluated the efficacy of intranasal FM administration in piglets undergoing castration.

Given the great potential of single or multimodal analgesia using FM and lidocaine in mitigating castration pain for swine, it is critical to further evaluate the efficacy of both drugs, particularly when administered utilizing less invasive administration techniques. Therefore, the objectives of this study were to determine the efficacy of buffered lidocaine administered intra-inguinally and FM given intranasally based on cortisol biomarker and UPAPS scoring on mitigating castration pain in piglets.

## 2. Materials and methods

This was a two-part study completed in the spring of 2022 on a commercial sow farm located in the Southeastern United States. This study was approved by the Institutional Animal Care and Use Committee of North Carolina State University (IACUC protocol 20-113-01). Animals were cared for and handled in accordance with the Guide for the Care and Use of Agricultural Animals in Research and Teaching (25). No animals were castrated exclusively for the purposes of this study, the piglets’ castration was a regular procedure conducted on the farm, that contributes to the four Rs of animal experimentation (reduce, replace, refine, and respect 26), and the welfare of pigs.

### 2.1. Housing and management

Piglets were housed with sows in fully slatted, tunnel-ventilated farrowing rooms. Room temperature was managed through a computerized control system at 22° ± 1.0° C for the sow and heat mats for piglets were set to approximately 30–35°C. Within each room, sows and litters were housed in individual farrowing crates (2.5 m × 0.7 m) with additional space for piglets (2.5 m × 1.3 m) surrounding the crates. Lighting was turned on between 600 h and 1,630 h. Feed and water were offered *ad libitum* to sows and piglets.

This study produced two data sets: one for Part I: physiological response and Part II: for behavioral assessment.

### 2.2. Part I: physiological assessment

#### 2.2.1. Treatment

**Figure 1:**
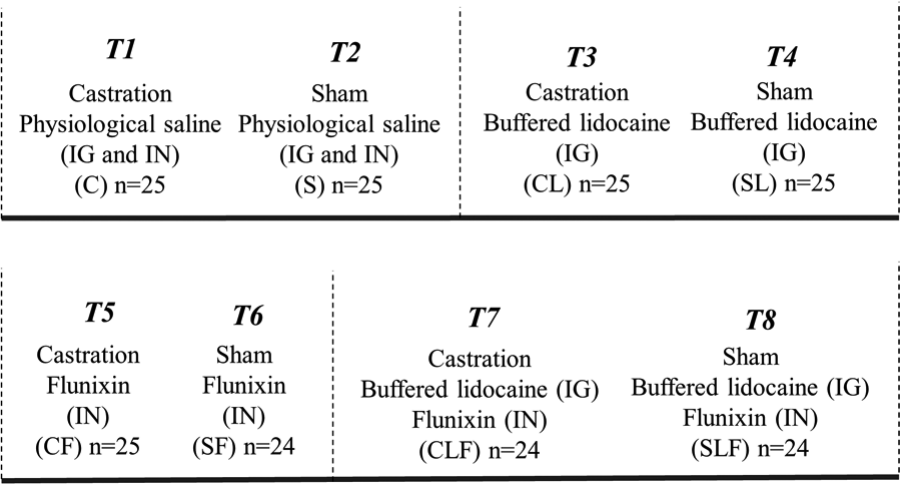
Treatment allocation for part I^3^. Procedure: surgical castration or sham castration; treatment: physiological saline or buffered lidocaine and/or flunixin (2.2 mg/kg); route of administration: Inguinal (IG) and/or Intranasal (IN).

A total of 197 Large White x Duroc cross male piglets from 35 litters were enrolled in the study (Table 1). Piglets were individually identified using ear tags (Allflex Global Piglet ear tags, Allflex Livestock Intelligence, Madison, WI), weighed, and randomly allocated to one of eight treatment groups (Figure 1).

**Table 1:** Mean ± SD. Descriptive statistics for 35 litters at enrollment (Part I; 197 piglets total).

Age (days)	9.0 ± 1.1
Sow parity	3.9 ± 1.3
Total born	14.3 ± 1.8
Liveborn	13.1 ± 1.5
Stillborn	0.7 ± 0.9
Mummies	0.5 ± 0.9
Weight (Kg)	3.2 ± 0.7

#### 2.2.2. Treatment administration

##### 2.2.2.1. Buffered lidocaine

Lidocaine was buffered by mixing 2 ml of 8.4% Sodium Bicarbonate with 20 ml of 2% lidocaine HCl injectable solution (20 mg/ml) to achieve a pH of 6.8 (Lidocaine Hydrochloride, Covetrus, Dublin, Ohio, US). Piglets enrolled in lidocaine treatment groups (CL, SL, CLF, SLF^1^) were injected with buffered lidocaine approximately 20 min prior to surgical castration using a dose approximately of 20 mg/kg. To the author's knowledge, there are no studies that have determined the effective dose concentration of lidocaine in neonatal pigs and extrapolating effective dose concentrations from other species like ruminants is inappropriate given unique physiological differences between species and age. Therefore, the dose was determined by the co-author (MSL) using over 3 years of extensive field experience implementing similar pain management during piglet castration. To date, over 40,000 piglets have been administered this dose of a local anesthetic as part of an on-farm protocol with no adverse effects noted due to the drug concentration.

Piglets were held by both rear legs by one caretaker with the abdomen facing the individual administrating treatment. Buffered lidocaine was injected intra-inguinally (Supplementary Image 1) by a second caretaker using a ½ inch needle (Ideal® D3 20 Gauge, Neogen, Lansing, MI) inserted into a syringe (Prima Tech® 2cc Bottle Mount Vaccinator, Prima Tech USA, Kenansville, NC). A total of 1.5 ml of buffered lidocaine per injection site was administered intra-inguinally (IG) into each inguinal canal (left and right) at a 40-degree angle 5 cm–7 cm from the scrotum and 2 cm–3 cm from the abdominal wall. Piglets enrolled in the control treatment (C, S, CF, SF^2^) were handled in an identical manner and 1.5 ml of sterile saline was injected in the same two locations as described previously. The inguinal injection was conducted while the piglets were awake and without sedation or anesthesia.

##### 2.2.2.2. Flunixin meglumine

Immediately following intra-inguinal injection, piglets enrolled in the FM treatment groups were held in sternal recumbency by one individual, and 2.2 mg/kg (Banamine®, Merck Animal Health, Madison, NJ, US) was administered in one nostril using a MAD® nasal intranasal mucosal atomization device (Telefex Incorporated, Wayne, PA, US) attached to a Prima Tech® 0.5cc bottle mount vaccinator. The same individual administered the treatment by gently holding the piglet's snout using their non-dominant hand to steady the head and administered the drug with the other hand. Piglets in the control group were handled in the same manner in an equivalent volume of 0.2 ml of sterile saline was administered as described above.

##### 2.2.2.3. Castration procedure

Castration was performed by one trained caretaker from the farm. Piglets were picked up, individually held by both hind legs with head down, and two vertical incisions were made through the skin of the scrotum over each testicle using a scalpel blade. Once the incisions were made, testicles were exposed, spermatic cords cut, and testicles were completely removed by traction. A sham castration was performed to mimic similar handling conditions in which piglets were picked up, held in the same manner, and had pressure applied to the scrotal area by the same individual responsible for castration.

##### 2.2.2.4. Blood sampling

**Figure 2:**
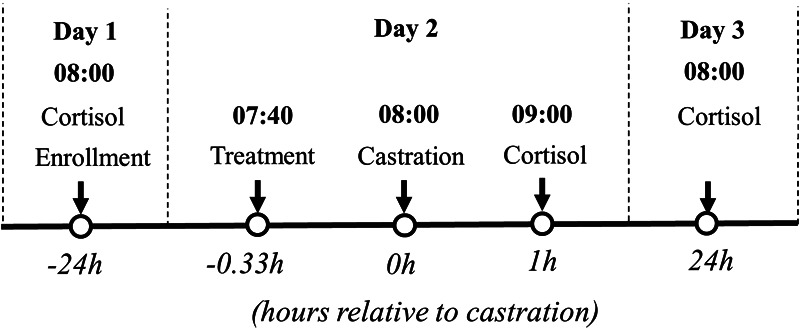
Flow chart of the study (part I) design based on hour relative to castration. Litters enrolled in the study were randomly assigned to: (1) surgical castration or sham castration; (2) treatment: physiological saline or buffered lidocaine and/or flunixin (2.2 mg/kg); (3) route of administration: Inguinal (IG) and/or Intranasal (IN).

Blood was collected 24 h prior to (−24 h), 1 h (1 h), and 24 h post castration (24 h, Figure 2). Blood samples were collected using the technique described in other studies (24, 27). The orbital sinus cavity was punctured using an Excel® disposable hypodermic needle 20G (Exel International, Quebec, Canada) and deposited into a 4 ml BD® red vacutainer serum tube (Med Vet International, Mettawa, IL). All tubes were maintained in a cooler and centrifuged (2,000 × g for 15 min at 4°C) no more than eight hours post-collection to separate serum. Serum was stored in 1.5 ml Axygen® microcentrifuge tubes (Axygen Scientific, Corning, NY) at −80°C and, assays were performed two months later.

##### 2.2.2.5. Cortisol assay

Serum cortisol concentrations were quantified using a commercially available EIA kit (Arbor Assays DetectX Cortisol EIA Kit, Product # K003). The detection limits of the cortisol assay were 50 pg/ml to 3,200 pg/ml. Samples were diluted 1:100 with assay buffer and run according to kit directions. All samples were assayed in duplicate. In total, forty cortisol assays were performed. The mean intra-assay variation of duplicate samples was 6.7% ± 7.5%. The mean inter-assay variation of the two quality control pools was 10.0% ± 0.1% (Merenda et al., 17).

### 2.3. Part II: behavioral assessment

Upon obtaining results from the physiological assessment of treatments in Part I, a follow-up behavioral study was conducted to assess the efficacy of lidocaine and FM in combination on mitigating castration pain in pigs using a validated piglet pain scale (24, 28, 29). Another cohort of piglets was enrolled in this second part of this study consisting of a total of 119 Large White x Duroc cross male piglets (60 and 59 piglets for C and CLF respectively, Table 2).

**Table 2:** Mean ± SD. Descriptive statistics for 16 litters at enrollment (Part II; 119 piglets total).

Age (days)	7.9 ± 0.9
Sow parity	3.5 ± 1.5
Total born	15.2 ± 3.3
Liveborn	13.8 ± 3.0
Stillborn	1.1 ± 1.2
Mummies	0.3 ± 0.4

#### 2.3.1. Behavioral scoring

**Figure 3:**
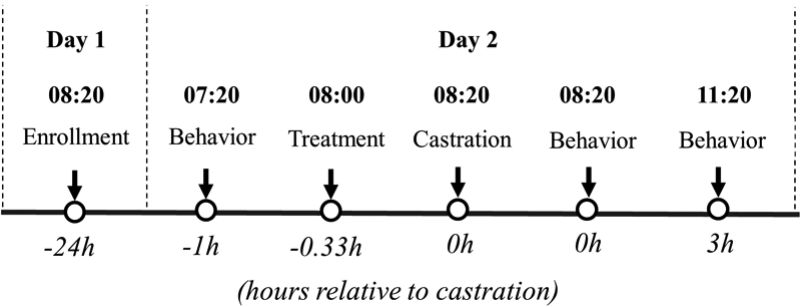
Flow chart of the study design (part II) based on hour relative to castration. Litters enrolled in the study were randomly assigned to: T1: (**C**) Castration + physiological saline (IG & IN) *n* = 30 and T2: (CLF) Castration + buffered lidocaine 2% (IG) Flunixin (IN) *n* = 29.

Behavior scoring was obtained in real-time by observing each piglet for 4-min continuously using Unesp-Botucatu pig acute pain scale (UPAPS, 29). Each piglet was scored by one trained observer at the following timepoints: 1 h before castration (−1 h), immediately post-castration (0 h), and 3 h post-castration (+3 h, Figure 3). The 4-min sampling time was obtained from the methodology previously validated (24, 28, 29). Treatments were masked, randomized, and applied to each piglet by a senior researcher.

The Unesp-Botucatu UPAPS scale evaluates five behavioral items, with each item divided into four descriptive levels (24, 28, 29). A numerical score was designated from “0” to “3”, with a “0” representing normal behavior (free of pain) and a “3” corresponding to pronounced behavioral deviation (severe pain). Therefore, for each timepoint, piglets may receive a score ranging from 0 (min) to 15 (max; Table 3). Total pain scores were then calculated for each piglet per timepoint.

**Table 3:** The UNESP composite pain scale (UPAPS) for scoring pain in piglets.

Item	Score	Score/criterion	Links to videos
Posture	0	Normal (any position, apparent comfort, relaxed muscles) or sleeping	https://youtu.be/QSosCD2SD4E
	1	Changes posture, with discomfort	https://youtu.be/SpaWsFCrPxE
	2	Changes posture, with discomfort, and protects the affected area	https://youtu.be/VjSlsRrG8yA
	3	Quiet, tense, and back arched	https://youtu.be/pm4hJ5163ao
Interaction and interest in the surroundings	0	Interacts with other animals; interested in the surroundings or sleeping	https://youtu.be/-880STgYq2I
	1	Only interacts if stimulated by other animals; interested in the surroundings.	https://youtu.be/nXjOdwn3dyw
	2	Occasionally moves away from the other animals, but accepts approaches; shows little interest in the surroundings	https://youtu.be/2k2JDr5U6As
	3	Moves or runs away from other animals and does not allow approaches; disinterested in the surroundings	https://youtu.be/se70oYXcWFw
Activity	0	Moves normally or sleeping	https://youtu.be/cC75t7L5-YA
	1	Moves with less frequency	https://youtu.be/lQo9wq8LAn8
	2	Moves constantly, restless	https://youtu.be/YQRJjijLvpk
	3	Reluctant to move or does not move	https://youtu.be/Zyx0G3Wpt8o
Attention to the affected area		A. Elevates pelvic limb or alternates the support of the pelvic limb	https://youtu.be/UD99ftO7HE0
		B. Scratches or rubs the painful area	https://youtu.be/7idfFk1harE
		C. Moves and/or runs away and/or jumps after injury of the affected area	https://youtu.be/u-Pqubom278
		D. Sits with difficulty	https://youtu.be/ETNEOCVV4h0
	0	All the above behaviors are absent	
	1	Presence of one of the above behaviors	
	2	Presence of two of the above behaviors	
	3	Presence of three or all the above behaviors	
Miscellaneous behaviors		A. Wags tail continuously and intensely	https://youtu.be/pU5dGZFNRHc
		B. Bites the bars or objects	https://youtu.be/cF3dsq7gMtk
		C. The head is below the line of the spinal column.	https://youtu.be/ZcIgngclRpI
		D. Presents difficulty in overcoming obstacles (example: another animal)	https://youtu.be/HlvdOI3lGuY
	0	All the above behaviors are absent	
	1	Presence of one of the above behaviors	
	2	Presence of two of the above behaviors	
	3	Presence of three or all the above behaviors	

### 2.4. Indication of analgesia

Following pain assessment scoring for each treatment, the observer was required, based on clinical experience, to mark whether the piglet indicated (yes) or did not indicate (no) a need for analgesic intervention (30). The analgesic intervention was not implemented following behavioral assessment and indication of analgesia was determined post-experiment when total counts were calculated and analyzed. Indication of analgesic need was assessed by treatment and timepoint and cutoff points were established using the collected data retrospectively.

### 2.5. Statistical analysis

Statistical significance was declared at *P* ≤ 0.05. All data were analyzed using RStudio (Version 4.1.0; 2021-06-29; RStudio, Inc., Boston, MA, USA, 31).

#### 2.5.1. Part I

A multilevel linear model was conducted with the cortisol concentrations after the Box-Cox transformation (*λ* = 0.02) to closely reassemble normality attested by Cramer-Von Mises test. Treatments, timepoints (−24 h, 1 h, 24 h), and their interaction were used as fixed effects. Piglet's age, sow parity, and piglet body weight were included as covariables. Piglets nested in the litter were applied as random effects composing each modeling level. The Bonferroni adjustment was used for the *P*-values and the Tukey method was utilized as *post hoc* test with statistical significance declared at *P* ≤ 0.05. Results were illustrated with boxplots using the original cortisol concentration values.

#### 2.5.2. Part II

A multilevel generalized linear model adjusted by Poisson distribution was used to analyze total pain score using treatments (C and CLF), timepoints (Baseline at −1 h, immediately post-castration, and post-castration at 3 h), and its interaction as fixed effects. Piglet's age and sow parity were included as covariables. Piglets nested in the litter were applied as random effects composing each modeling level. The Bonferroni was used for adjustment after multiple comparisons to the *post hoc* test. Results were illustrated with boxplots.

For indication of analgesia based on the evaluator's clinical experience and based on UPAPS's cutoff point (total sum ≥4), a test of homogeneity by Chi-square (*χ*^2^) was used to determine if the distribution of the piglets indicating the need for analgesic intervention was the same between the two treatments (C and CLF) for each timepoint and the entire period.

## 3. Results

### 3.1. Part I

Data were collected from a total of 197 male piglets over 35 litters with 5.6 ± 1.7 piglets enrolled per litter. Piglet and litter performance can be found in Table 1.

### 3.2. Effect of the treatment and timepoint on cortisol concentrations

Treatment (*P *< 0.05), timepoint (*P *< 0.05), and the interaction treatment by timepoint (*P *< 0.05) had an effect on cortisol concentrations. Age (*P* = 0.70) and sow parity (*P* = 0.44) had no effect on the cortisol concentration, while the piglet body weight had a negative (*β* = −0.06) and significant (*P *< 0.05) effect.

Average cortisol concentrations did not differ at −24 h (*P* > 0.05) or at 24 h post-castration (*P *> 0.05) between treatments. At 1 h post-castration, castrated piglets (C and CL) demonstrated greater cortisol concentrations than piglets assigned to sham treatment groups (S, SF, SL, SLF; *P *< 0.01). Cortisol concentrations between C and CL at 1 h post-castration were not different (*P* > 0.05).

**Figure 4:**
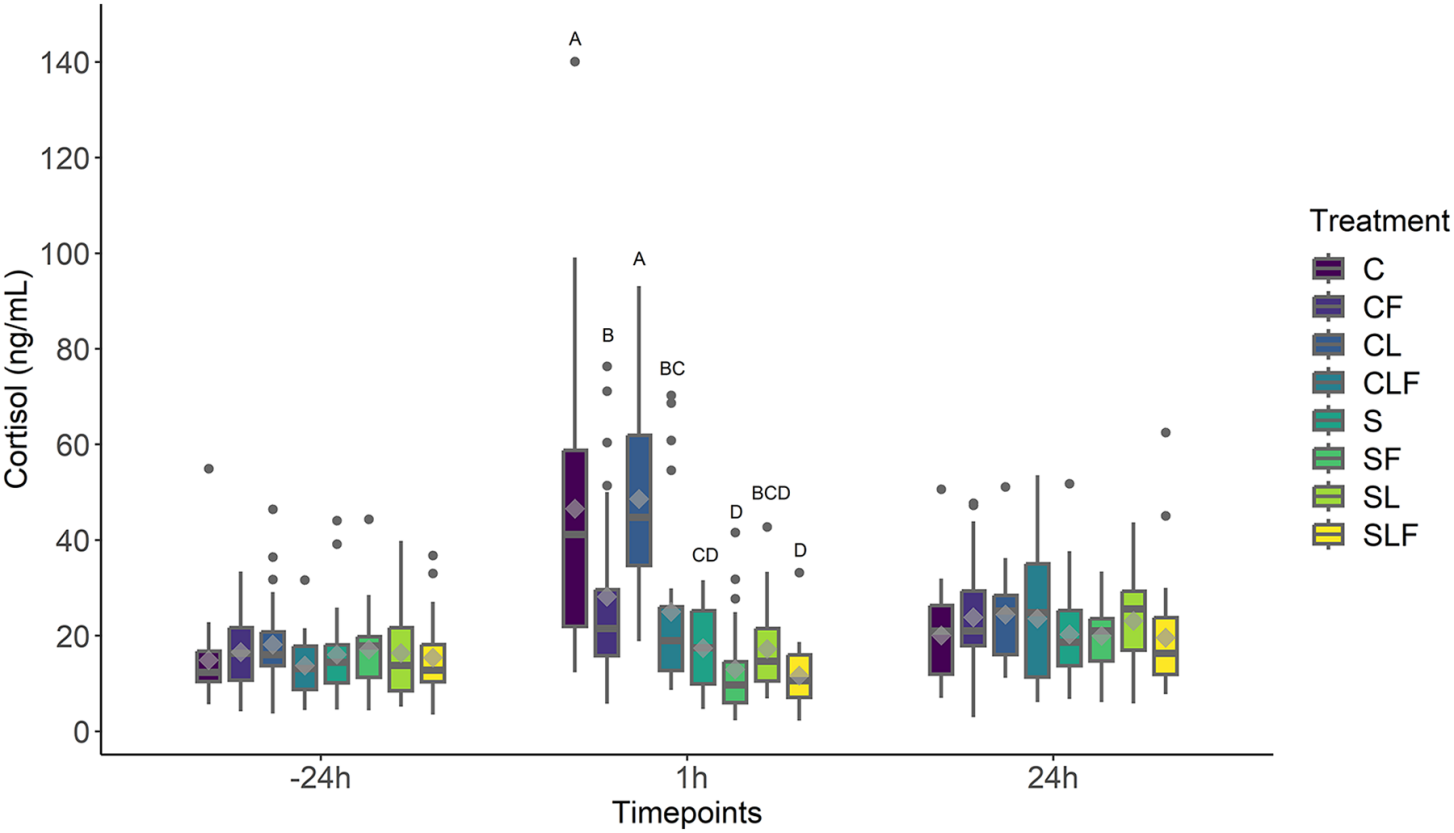
Boxplot of cortisol concentrations (ng/ml) for piglets in the C, S, CL, CF, SF, SL, CLF and SLF groups^4^ over three timepoints. Timepoint (*P *< 0.01), treatment (*P *< 0.01) and treatment by timepoint (*P *< 0.01) effect. Symbols: circle • indicates outliers; diamond ♦ indicates the mean. Different capital letters show differences statistically significant (*P *≤ 0.05) where A > B > C > D.

Castrated piglets in the CF and CLF group had lower cortisol concentrations compared to C and CL-treated pigs (*P* < 0.05). Sham piglets (S) demonstrated lower cortisol concentrations compared to CF piglets (*P* < 0.05) but were not different compared to CLF-treated piglets (*P* > 0.05). Sham piglets treated with FM (SF and SLF) had the lowest cortisol concentrations and were different than all castrated piglets (*P *< 0.05). No differences were found between any sham treatment group at any timepoint (*P* > 0.05, Figure 4).

### 3.3. Part II

Data were collected from 16 litters with a total of 119 male piglets with 3.8 ± 0.8 piglets enrolled per litter. Piglet and litter performance can be found in Table 2.

### 3.4. Effect of the drug, procedure, and timepoint on total pain scores

**Figure 5:**
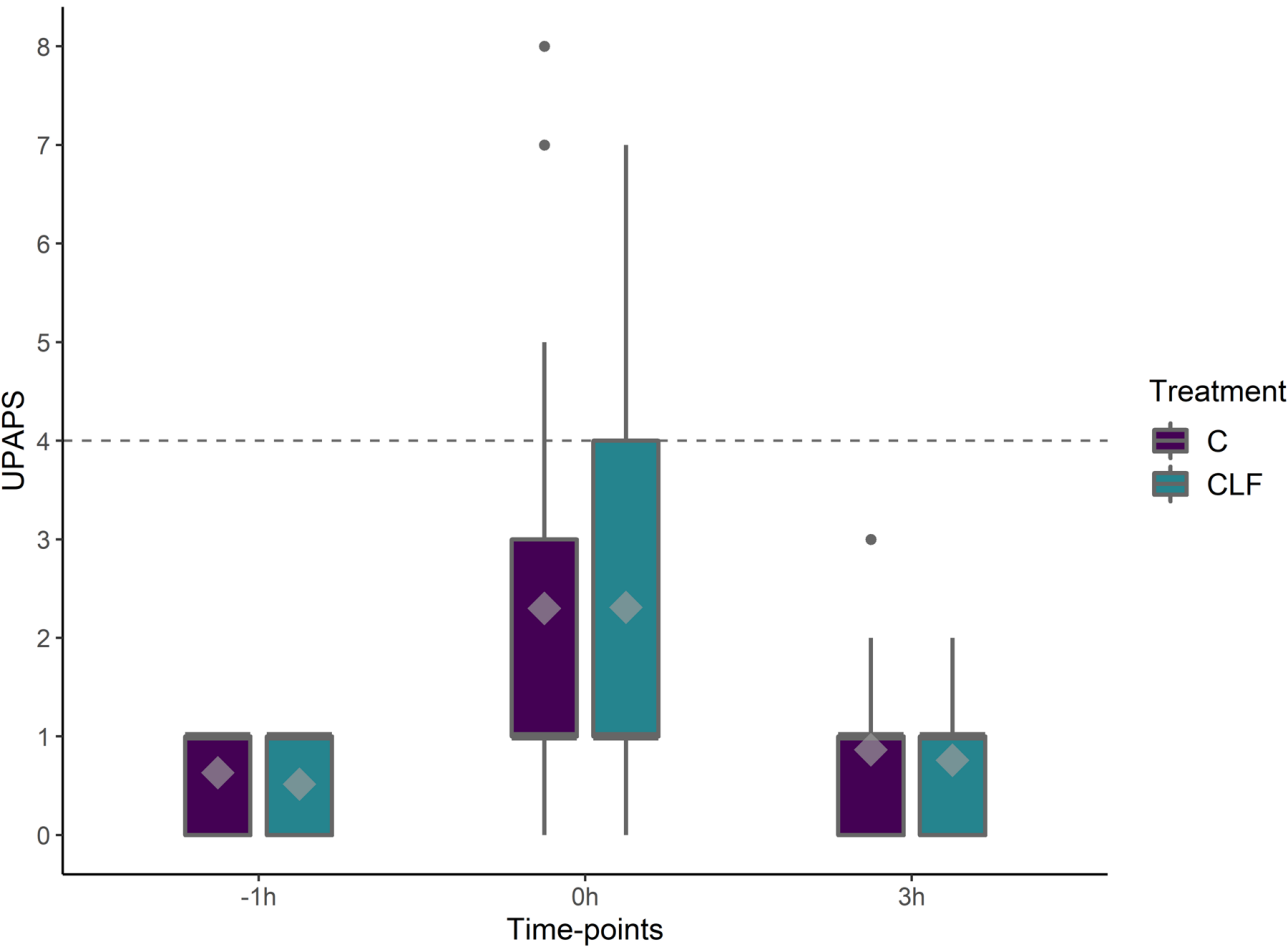
Boxplots of UPAPS (Unesp-Botucatu Pig acute pain scale) for piglets in the C and CLF groups over three timepoints. Timepoint (*P *< 0.01), treatment (*P *< 0.01) and treatment by timepoint (*P *< 0.01) effect. Symbols: circle (•) indicates outliers; diamond (♦) indicates the mean; the horizontal gray dashed line indicates the UPAPS's optimal cut-off point (≥4). Different capital letters show differences statistically significant (*P *≤ 0.05) where A > B > C > D.

There was a timepoint (*P* < 0.05) effect on UPAPS with total average pain scores greatest immediately post-castration compared to pre-castration timepoint. Piglet age (*P* = 0.06) and sow parity (*P* = 0.5) had no effect on the UPAPS. There were no differences between treatment or treatment by timepoint (*P* > 0.05, Figure 5).

When assessing indication of analgesic requirement based on evaluator clinical experience, the treatment C (*n* = 90) and CLF (*n* = 97) was not different at timepoint −1 h (C and CLF respectively 0 vs. 0; *χ*^2^ = 0.01, *P *= 0.9), 0 h (C and CLF respectively 8 vs. 8; *χ*^2^ = 0.00, *P* = 1.0), and 3 h (C and CLF respectively 0 vs. 0; *χ*^2^ = 0.01, *P *= 0.9) or in all timepoints (C and CLF respectively 8 vs. 8; *χ*^2^ = 0.00, *P* = 1.0).

When assessing indication of analgesic requirement based on UPAPS's cutoff point (total sum ≥4), the treatment C (*n* = 90) and CLF (*n* = 97) were not different at timepoint −1 h (C and CLF respectively 0 vs. 0; *χ*^2^ = 0.01, *P *= 0.9), 0 h (C and CLF respectively 7 vs. 8; *χ*^2^ = 0.001, *P* = 0.94), and 3 h (C and CLF respectively 0 vs. 0; *χ*^2^ = 0.01, *P *= 0.9) or in all timepoints (C and CLF respectively 7 vs. 8; *χ*^2^ = 0.005, *P* = 0.95).

## 4. Discussion

Castration is a common procedure performed on farm despite ethical concerns specific to pain experienced by the piglet. Pain mitigation strategies in the US are limited with most of the work assessing the efficacy of local anesthesia and NSAIDs in controlling castration pain. Pain management protocols should be implemented in a manner that is effective, practical, cost-effective and the least invasive for the piglets. Therefore, the objectives of this study were to determine the efficacy of buffered lidocaine administered intra-inguinally based on cortisol biomarker and FM administered intranasally using UPAPS on mitigating castration pain in piglets.

### 4.1. Physiological response

The pioneering spirit of the present study was the use of intranasal FM to mitigate castration stress in piglets as demonstrated by decreased cortisol concentrations immediately following castration. This finding agrees with research previously conducted in 2021 by Nixon and colleagues that evaluated intramuscularly administered FM efficacy on the castration stress response. Results from the 2021 study proved that FM decreased cortisol concentrations 2 h post-castration when compared to a castrated, non-treated control group (32). In addition, although not significant, cortisol concentrations were also found to numerically decrease by more than 30% in piglets administered FM topically 24 h prior to castration compared to saline-treated piglets (17).

On the other hand, sham piglets treated with intranasal FM showed the greatest cortisol reduction when compared with CLF group, this might be an indication that FM was able to decrease the stress produced by handling. This aligns with Nixon and colleagues (32), FM demonstrated the greatest cortisol reduction in sham and FM-treated piglets. In another study (33) concluded that the extra-time involving the administration of local anesthesia may increase the stress and discomfort due to the double handling.

This is the first paper utilizing an intra-inguinal approach to administering lidocaine as a local anesthetic, targeting direct inhibition at the spermatic cord. Buffered lidocaine administered intra-inguinal had no effect on stress mitigation from a physiological response standpoint. The results from this study agree with numerous studies that have consistently demonstrated lidocaine does not decrease cortisol concentrations in castrated piglets (34–36) and in fact may increase cortisol concentrations when compared to castrated piglets receiving no anesthetic (37, 38). However, past work conducted (39, 40), and more recent studies in 2022 (41) have demonstrated lidocaine efficacy in mitigating castration stress. There seems to be no consensus in the literature about the effectiveness of lidocaine in reducing the physiological responses in piglets undergoing castration, however, this can be explained by differences in the interval between treatment administration and castration (0, 3, 5, 10, or 20 min). While buffering the lidocaine provided the advantage of preventing pain associated with the injection site, future studies must consider the refining injection technique to ensure spermatic cord innervation is impacted and administration can be consistently given across pigs regardless of the differences in anatomical structures.

Results from the current study and support from the previously published work suggest that FM's mode of action is effective in mitigating deviations to the physiological response of piglets undergoing castration as determined by decreased cortisol levels immediately following the procedure.

### 4.2. Behavioral response

In contrast to the physiological response to castration, piglets administered FM intranasally did not decrease total behavior pain scores and indication of analgesic need was similar between treated control piglets. The present work is in direct contrast with previous work (24) that showed transdermal FM administered 24 h before castration decreased total pain scores and indication of analgesic need from 54% (control pigs) to 29% (transdermal FM treated pigs). There are several possible explanations for this, including drug absorption variability and behavioral methodology. From a drug absorption standpoint, intranasal administration is often characterized as a rapid route for drug absorption given the nasal mucosa is richly supplied with blood vessels and intranasal administered drugs gain immediate access to systemic circulation (42). Furthermore, intranasally administered products, as opposed to topically applied products, may bypass the hepatic first-pass effect, thus altering both the concentration and time in which the drug reaches the maximum concentration in the blood (43). Therefore, moments in which total pain scores and indication of analgesic need were assessed in this study may have been influenced by varying absorption time between administration routes thus pain scores may have been assessed when the drug was not at peak efficacy, resulting in non-significant differences between control and treated pigs. Future work must assess pharmacokinetic and pharmacodynamic properties of FM administered intra-nasal to identify C_max_ and T_max_ more effectively for behavioral research.

In addition to absorption variability, behavioral methodology may have also influenced overall results of this study. The validated pain scale effectively distinguished painful and non-painful states in castrated piglets as observed via deviations in total pain scores across timepoints, however, treatment was not different. In contrast to Lopez-Soriano and colleagues (24), total pain scores and indication of analgesic need were evaluated via live observation as compared to video observation due to farm logistics. Work evaluating piglet behavior has demonstrated that pigs are prey species and will often hide behaviors specific to pain and injury (44–46). When comparing total pain scores immediately post castration in this study compared to (24), it should be noted that total scores were 4.9 for castrated piglets and 3.1 for transdermal flunixin treated piglets in contrast with the present study that the total pain score were approximately 2.3 for both C and CLF. Work conducted in rabbits (47) concluded that the presence of an observer might mislead to a false sense of pain. Therefore, future studies should evaluate total pain scores and indication of analgesic need utilizing recorded video, thus eliminating the impact of human presence on piglet's pain demonstration.

Based on the behavioral assessments, inguinal buffered lidocaine was not able to reduce the UPAPS scores between (CLF) treated piglets and piglets treated with physiological saline (C). As beforementioned, results of the efficacy of lidocaine are inconclusive as recently found (48) where a reduction but no complete elimination of the expressed pain-associated behaviors after local anesthesia was reported, however, lidocaine seemed to reduce the pain-associated behavior for a longer period compared to other local anesthetics.

## 5. Limitations

### 5.1. Physiological response

Physiological responses, including cortisol, were commonly used in previous studies as an indirect biomarker of pain in piglets following NSAID administration. Stress, handling, and mechanical stimuli (incision in the scrotal skin) might induce cortisol release (49). In pigs, cortisol levels may vary during the day and can also be affected by the type of breed (50).

### 5.2. Behavioral response

Intra-inguinal injection is not a routine procedure performed on farm and variation exists in injection site location based on pig size, needle size, piglet's position, and individual technique can be understood as a limitation of this study. Although only one person injected all pigs for the study, it is possible that injection technique was inconsistent, thus resulting in UPAPS scores variability of anesthetic efficacy.

## 6. Animal welfare implications and conclusions

### 6.1. Physiological response

This research was the first to measure the efficacy of inguinal buffered lidocaine administered intra-inguinal in combination with intranasal administered FM. Intranasal FM was able to effectively reduce the physiological response of piglet to castration as demonstrated by decreased cortisol levels immediately post-castration. Hence, from a husbandry view, the implementation of intranasal FM could be an important and feasible step to be applied in large-scale swine farms that normally do not use any drug for pain relief associated with surgical castration.

Cortisol concentrations were greater 24 h post-castration compared to baseline concentrations suggesting castrated piglets are still experiencing pain sensitivity one day following castration and a single FM administration was not effective in mitigating post-operative pain. Long-term research projects should focus on refining injection technique for inguinal buffered lidocaine and consider administration frequency and dosing of intranasal FM to control pain for a longer period post-castration.

### 6.2. Behavioral response

Inguinal buffered lidocaine did not reduce the behavioral response to pain in piglets undergoing castration. Further, studies are needed in obtaining a consistent methodology to administer inguinal buffered lidocaine and reducing the effects of human interaction during behavioral assessments.

This work has supported the continued drive to improve on-farm pig welfare by addressing the need for FDA-approved products to mitigate pain both pre and post-operatively. In this study, rescue analgesics were not administered as they were not part of the approved standard operating procedure for this commercial farm and the researchers did not have an established veterinary client patient relationship (VCPR). Administering a rescue analgesic without an established VCPR has both legal and food safety implications in the US and therefore, rescue analgesic was not administered. Future studies will include direct involvement of the attending veterinarian to ensure rescue analgesia can be administered to those animals demonstrating an indication of analgesia need.

## Data Availability

The raw data supporting the conclusions of this article will be made available by the authors, without undue reservation.

## Footnotes

^3^Treatment allocation for part II. Behavioral scoring using UPAPS for T1: (C) Castration+physiological saline (IM & IN) *n* = 30 and T7: (CLF) Castration+buffered lidocaine 2% (IM) and Flunixin (IN, 2.2 mg/kg) *n* = 29.

^1^**T3**: (CL) Castration plus buffered lidocaine (IG; *n* = 25); **T4**: (SL) Sham plus buffered lidocaine (IG; *n* = 25); **T7**: (CLF) Castration plus buffered lidocaine (IG) and flunixin (IN; *n* = 24); **T8**: (SLF) Sham plus buffered lidocaine (IG) and flunixin (IN; *n* = 24).

^2^**T1**: (C) Castration plus physiological saline (IG and IN; *n* = 25); **T2**: (S) Sham plus physiological saline (IG and IN; *n* = 25); **T5**: (CF) Castration plus flunixin (IN; *n* = 25); **T6**: (SF) Sham plus flunixin (IN; *n* = 24).

^4^T1: (C) Castration plus physiological saline (IM and IN; *n* = 25); T2: (S) Sham plus physiological saline (IM and IN; *n* = 25); T3: (CL) Castration plus buffered lidocaine (IM; *n* = 25); T4: (SL) Sham plus buffered lidocaine (IM; *n* = 25); T5: (CF) Castration plus flunixin (IN; *n* = 25); T6: (SF) Sham plus flunixin (IN; *n* = 24); T7: (CLF) Castration plus buffered lidocaine (IM) and flunixin (IN; *n* = 24); T8: (SLF) Sham plus buffered lidocaine (IM) and flunixin (IN; *n* = 24).
